# Effects of Vitamin D and Calcium Supplementation on Micro-architectural and Densitometric Changes of Rat Femur in a Microgravity Simulator Model

**DOI:** 10.5812/ircmj.18026

**Published:** 2014-06-05

**Authors:** Marjan Kouhnavard, Ensieh Nasli Esfahani, Mohammad Montazeri, Seyed Jafar Hashemian, Mitra Mehrazma, Bagher Larijani, Amir Nezami Asl, Amir Khoshvaghti, Ammar Falsafi, Komeil Lalehfar, Keyvan Malekpour, Mehran Vosugh

**Affiliations:** 1 Diabetes Research Center, Endocrinology and Metabolism Clinical Sciences Institute, Tehran University of Medical Sciences , Tehran, IR Iran; 2 Endocrinology and Metabolism Research Center, Endocrinology and Metabolism Research Institute, Tehran University of Medical Sciences, Tehran , IR Iran; 3 Young Researchers Club, Babol Branch, Babol, IR Iran; 4 Oncopathology Research Center, Iran University of Medical Sciences, Tehran, IR Iran; 5 Aerospace and Subaquatic Medicine Faculty, AJA University of Medical Sciences, Tehran, IR Iran

**Keywords:** Weightlessness, Bone Density, Osteoporosis, Dietary Supplements, Calcium, Vitamin D

## Abstract

**Background:**

Revealing data on the role of vitamin D and calcium supplementation in bone health has led some to suggest that vitamin D and calcium treatment could also play a role in protecting bone against microgravity-induced mineral loss.

**Objectives:**

The aim of the present study was to investigate the effects of vitamin D and calcium administration on microscopic and densitometric changes of rat femur in a Microgravity Simulator Model.

**Materials and Methods:**

After designing a Microgravity Simulator Model, 14 rats were placed in the cages as follows: seven rats as osteoporosis group and seven rats received oral supplement of calcium/vitamin D as the treatment group. Animals were sacrificed after eight weeks and then both femurs were removed. Bone mineral density was measured for one femur from each animal, and morphologic studies were evaluated for the contralateral femur.

**Results:**

Bone mineral density of the whole femur in the treatment group was significantly higher than the osteoporosis group (0.168 ± 0.005 vs. 0.153 ± 0.006, P = 0.003). Also, bone mineral content of the whole femur was significantly higher in treatment group (0.415 ± 0.016 vs. 0.372 ± 0.019, P = 0.003). However, resorption eroded surface percentage was higher in the osteoporosis group (18.86 ± 3.71% vs. 9.71 ± 1.61%, P = 0.002).

**Conclusions:**

According to the results of this study, vitamin D and calcium administration might have protective effects against microgravity-induced mineral loss in a Rat Microgravity Simulator Model.

## 1. Background

Bone loss during spaceflight is about 1–2% per month (1). Bone mass changes are, however, site-specific rather than evenly distributed throughout the skeleton with the tendency that weight bearing bones are more affected by microgravity than non-weight-bearing bones (1, 2). In normal bone there is equilibrium between bone formation and bone resorption. Recent biochemical data from astronauts confirmed the previous findings in rats that microgravity induces an uncoupling of bone remodeling in regards to bone formation and resorption that could account for bone loss (3). Of the many types of bone loss countermeasures evaluated to date, none have been proven effective during sapceflight (4). The space environment itself results in physiologic changes that can alter nutritional status. Vitamin D is a nutrient linked to the bone health and as such has been suggested as a potential countermeasure (5). Regardless of its efficacy in preventing bone loss, if the vitamin D status of astronauts were established as suboptimal before the spaceflight, vitamin D supplementation might be warranted (6-8). Also space flight-induced bone and calcium loss has been documented for decades, and has been the subject of many reviews (9, 10). When bone loss is considered, calcium intake is an obvious initial concern. Based on reviews of available information, a decision was made requiring that the International Space Station food system shall provide 1000–1200 mg calcium per day, similar to the recommendations for Earth-based conditions (10). The role of the calcium endocrine system in this response of bone tissue has been studied through the analyses of blood samples acquired during ground-based simulations and spaceflight (11, 12). Detailed listing of human spaceflight results revealed that the two major calcemic hormones, parathyroid hormone and 1,25-dihydroxyvitamin D, were suppressed and that calcitonin was unchanged (12). The increase in calcium levels due to limb unloading might not be detectable through laboratory tests since it might be kept in normal ranges. However, this increase in serum calcium results in a decrease in parathyroid hormone (12). Data emphasizing a role for vitamin D and calcium supplementation in bone health on Earth has led some to suggest that vitamin D and calcium treatment could play a role in protecting bone against microgravity-induced mineral loss.

## 2. Objectives

The aim of the present study was to investigate the effects of vitamin D and calcium administration on microscopic and densitometric changes of rat femur in a hindlimb suspension model.

## 3. Materials and Methods

This is an experimental study approved by the Ethics Committee of Animal Research at AJA University of Medical Sciences (approval code: 991176 on 8 February 2012).

### 3.1. Animals

Adult male rats were obtained from the Experimental Animal Facility of Tehran University of Medical Sciences. All animals were kept under the same laboratory condition at 21 ± 1°C and 12:12 hours light/dark cycle. Equal number of rats (n = 14) were utilized for osteoporosis and treatment groups. All rats were allowed free access to laboratory food pellets and fresh clean drinking water at all time. Treatment group received calcium/vitamin D containing diet as 100 mg/kg calcium carbonate and 40 IU/kg cholecalciferol in their daily water. Care for the animals used in the experiments exceeded the standards set forth by the National Institutes of Health in their guidelines for the care and use of experimental animals.

### 3.2. Microgravity Simulator Model

**Figure 1.**
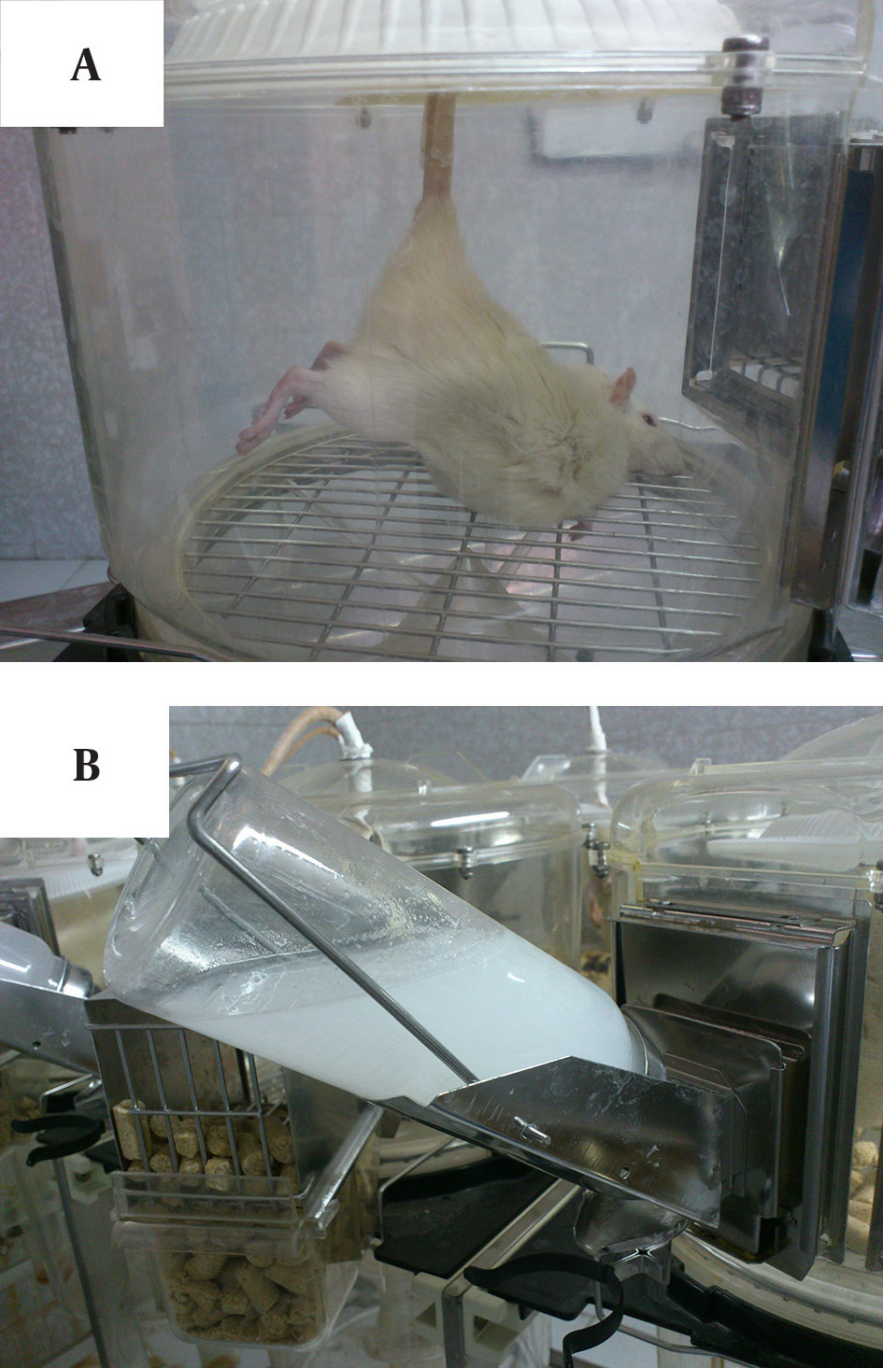
A: Hindlimb suspended rat in a metabolic cage. Rat’s body makes an angle of 30 degrees with the cage floor. B: Supplementation with calcium/vitamin D in daily water.

Hind limb unloading was performed using a microgravity simulator model as described previously (13). Briefly, the rats were first anesthetized by an intra-peritoneal injection of Ketamin (80 mg/kg) and Xylazine (5 mg/kg). Then the rats were gently wrapped in a towel. The tail of the rats were then passed through the hole located in the cage inlet (as shown in Figure 1). The tail was cleaned by ethanol and all the dead and dirty skin was removed. Then it was allowed to dry which took one minute or less. Afterwards, the traction tape was adhered to the tail exactly from the base of the tail just above the hair line. The traction tape was narrow enough so that the tape on one side of the tail did not come into contact with the tape on the other side of the tail. A propylene piston which was cut in a longitudinal pattern and divided into two equal parts was put on the traction tape. The two parts of the pistons were secured by two transverse filaments (length: 3.8 mm, width: 6 mm). These transverse filaments were placed on both sides of the pistons. They were tightly attached in order to avoid the detachment of the traction tape. However, they were loose enough to allow normal blood circulation to the tail.

Then a supporting disc was stuck to the base of the pistons to avoid their detachment by the rats. Because the rat’s tail plays an important role in the regulation of the body temperature, only some areas of the rat’s tail were covered by the traction tape and the rest was exposed to the air. After suspending the rats, transverse processes of the pistons in the ending sides were stuck to the inlet of the metabolic cage to avoid detachment of the rats. In addition, since the piston surface was smooth, the unloading apparatus imparted minimal resistance so that the rats were able to move in all directions in the cage.

Rat’s body made an angle of 30 degrees with the cage floor (14). Therefore, the hind limbs did not touch the cage floor. This angle as well as rat’s height was measured daily in order to be rearranged if necessary. In this model, rats had free access to water and food.

Rats were examined twice a day by an attending veterinarian to evaluate their overall appearance and activity as well as their eating and drinking status, ability to move freely and their tail condition.

### 3.3. Tissue Preparation

For measurements, rats were anaesthetized with sodium pentobarbital (50 mg/kg IP) and sacrificed by cervical dislocation. The femurs were removed and were prefixed with PLP fixative (2% paraformaldehyde containing 0.075 M lysine and 0.01 M sodium periodate solution, pH 7.4, stored at 4°C). These were then subsequently demineralized with 10% (wt/vol) EDTA and dehydrated with increasing concentration of alcohol concentrations before being embedded in paraffin. Low melting paraffin was used for alkaline phosphatase (ALP) staining. Then the paraffin blocks were placed in a microtome and 3.5 μm transverse sections were obtained.

### 3.4. Assays for Bone Mineral Density (BMD) and Bone Mineral Content (BMC)

BMD and the relative BMC of the femurs were measured using dual-energy X-ray absorptiometry (DEXA). This method has been described previously (15).

### 3.5. Histomorphometric and Statistical Study

The histomorphometric parameters were defined according to the report by the American Society for Bone and Mineral Research Committee. It was performed using Image Analyser in the Histology Department, Tehran University of Medical Sciences:

1. Outer cortical bone thickness (μm): mean width of outer cortical bone. This was measured by drawing a perpendicular line from the periosteum to the endosteum (16),
2. Trabecular bone volume (%): percentage of cancellous bone area occupied by trabeculae and expressed as percentage of total measured area (area of trabeculae and bone marrow space) (17),
3. Osteoid thickness (μm): mean thickness of the osteoid layer overlying the bone trabeculae (18),
4. Relative osteoid surface (%): percentage of cancellous bone surface with osteoid, including surfaces with and without osteoblasts (17),
5. Relative bone resorption eroded surface (%): percentage of cancellous bone surface with characteristic features of resorption, including surfaces with and without osteoclasts (17).

### 3.6. Statistical Analysis

All data were analyzed by Statistical Package for Social Studies (SPSS) version 17 (SPSS Inc. Chicago, IL, USA). Data are presented as mean (±SD) in the Tables. To compare the mean of variables between two groups, Mann-Whitney test was applied. Correlation was done using Spearman rank correlation. P-values < 0.05 were considered statistically significant.

## 4. Results

In this study none of the rats slipped out from the suspension apparatus prior to the completion of the study. No signs of local or systemic inflammation were observed throughout the study. Table 1 shows the means and standard deviation numbers of outer cortical bone thickness, trabecular bone volume, osteoid thickness, osteoid surface percentage, and resorption eroded surface percentage in the two studied groups.

Trabecular bone volume was higher in the treatment group compared with the osteoporosis group. However, osteoid surface percentage and resorption eroded surface percentage were higher in the osteoporosis group. In the osteoporosis group, resorption eroded surface percentage correlated with trabecular bone volume (r = -0.91, P = 0.006) and osteoid thickness (r = 0.77, P = 0.041). But in the treatment group resorption eroded surface percentage correlated with osteoid surface percentage (r = -0.81, P = 0.025). Bone mineral density and bone mineral content of proximal, mid, distal and whole femur in osteoporosis group were significantly lower than the treatment group (Tables 2 and 3).

**Table 1.** Comparison of the Means of all Parameters Measured in Two Different Studied Groups ^a^

	Osteoporosis	Treatment	P Value
**Outer cortical bone thickness, mm**	257.71 ± 40.93	307.86 ± 70.72	0.180
**Trabecular bone volume, %**	23.57 ± 4.68	39.29 ± 4.57	0.002
**Osteoid thickness, mm**	16.43 ± 4.39	12.71 ± 3.35	0.158
**Osteoid surface percentage, %**	62.43 ± 5.22	52.43 ± 7.59	0.005
**Resorption eroded surface percentage, %**	18.86 ± 3.71	9.71 ± 1.61	0.002

^a^ The data presented as mean ± SD. n = 7 for each group. P value analyzed with mann-whitney test.

**Table 2.** Comparison of the Means of Femur BMD (g/cm^2^) in Osteoporosis and Treatment Groups ^a^

	Osteoporosis	Treatment	P Value
**Whole femur**	0.153 ± 0.006	0.168 ± 0.005	0.003
**Proximal femur**	0.148 ± 0.006	0.163 ± 0.004	0.002
**Mid-femur**	0.151 ± 0.008	0.169 ± 0.006	0.003
**Distal femur**	0.159 ± 0.005	0.174 ± 0.005	0.003

^a^ The data presented as mean ± SD. n = 7 for each group. P value analyzed with mann-whitney test.

**Table 3.** Comparison of the Means of Femur BMC (gr) in Osteoporosis and Treatment Groups ^a^

	Osteoporosis	Treatment	P Value
**Whole femur**	0.372 ± 0.019	0.415 ± 0.016	0.003
**Proximal femur**	0.129 ± 0.005	0.144 ± 0.006	0.018
**Mid-femur**	0.111 ± 0.009	0.123 ± 0.006	0.003
**Distal femur**	0.132 ± 0.005	0.147 ± 0.005	0.004

^a^ The data presented as mean ± SD. n = 7 for each group. P value analyzed with mann-whitney test.

## 5. Discussion

During the space flight physiologic changes occur in the bone metabolism of astronauts, in response to microgravity including a decrease in circulating concentrations of the active form of vitamin D (1,25-dihydroxyvitamin D) and a consequent decrease in intestinal calcium absorption which finally results in the release of calcium from bones (18-21). Consequently, calcium is excreted through the gastrointestinal system and urine (22, 23).

In our study, BMD and BMC of rat femurs were significantly higher in the treatment group. The feeding levels of dietary calcium and vitamin D were in consistent with the previously reported study regarding tail suspension model in mice (24). Our results show that outer cortical bone thickness and trabecular bone volume were higher in the treatment group than in the osteoporosis group. In contrast, osteoid thickness, osteoid surface percentage and resorption eroded surface percentage were higher in the osteoporosis group which confirmed that an adequate vitamin D and sufficient dietary calcium result in a positive calcium balance, and consequently maximum bone architecture and strength is achieved (18). Each of the major bone cells such as osteoblasts, osteoclasts and osteocytes have the ability of metabolizing vitamin D to 1,25 vitamin D to exert biological activities including enhancement of maturation and mineralization by osteoblasts and osteocytes and reduction of bone resorption by osteoclasts (25). This shows that unloading might lead to a decrease in serum levels of 1, 25 vitamin D. Hence, vitamin D supplementation results in the recovery of bone formation, suggesting a correlation between the bone response to unloading and vitamin D levels (26, 27). It has been documented that vitamin D supplementation can significantly increase bone mass and decrease the risk of bone fracture (12, 28, 29). Kumei et al. revealed that microgravity can alter gene expression, where stromal cells of 1, 25 vitamin D treated rats had altered gene expression despite the presence of microgravity conditions (30).

Previous studies, also, showed that unloading reduces the fat-free weights of the tibia and lumbar vertebra, in comparison to the humerus and cervical vertebra which are not affected (31). The decrease in fat-free weights of unloaded bones relative to the controls is in concordance with the reduction in bone calcium content (27, 31). Another experimental study revealed that high dietary intake of calcium has a counteracting effect on the decreased bone mass caused by unloading (32). As shown in our study, Globus et al. also, demonstrated that high dietary calcium increases bone mass and mineral content in unloaded bones of the hindlimb unloaded rat (32).

Despite the promising results from experimental studies in the literature, there are limited data on interventions to stabilize bone metabolism and prevent bone loss in astronauts during space flight. It is suggested that a high calcium intake and vitamin D supplementation during space flight prevents an elevation of serum calcium levels through increased calcitriol levels and subsequent increased intestinal calcium absorption (33, 34). However, there are not enough data to prove the efficacy of pharmaceutical prevention of osteoporosis development in astronauts (7, 25). During space flight, due to inadequate sunlight (ultraviolet light) exposure and consequent vitamin D deficiency and low calcium intake, calcium and vitamin D supplementation may be a reasonable intervention for the prevention of bone loss (20, 34), although other studies have shown that high calcium intake and vitamin D supplementation during the space flight could not counteract the increase in bone resorption and the decrease in bone formation and consequently do not prevent the development of osteoporosis (33-35).

However, more studies with larger sample sizes should be done to prove that vitamin D and calcium supplementation can prevent cortical bone loss caused by unloading in hind limb suspended rats. We assume that daily calcium and vitamin D supplementation during a space flight might be essential to stabilize calcium balance and bone metabolism in order to prevent bone loss because of their potent anti-resorptive and anabolic effects on cortical and cancellous bone. In this study, we did not measure biomarkers for the osteoblastic activity, future studies should be performed to investigate the changes in serum biomarkers after such intervention. In conclusion, our study demonstrated that daily supplementation with calcium and cholecalciferol could improve BMD, BMC and morphologic changes in rats under microgravity conditions and could protect bones against microgravity-induced mineral loss in a rat microgravity simulator model.
